# Enhancing malaria diagnosis through microfluidic cell enrichment and magnetic resonance relaxometry detection

**DOI:** 10.1038/srep11425

**Published:** 2015-06-17

**Authors:** Tian Fook Kong, Weijian Ye, Weng Kung Peng, Han Wei Hou, M Marcos, Peter Rainer Preiser, Nam-Trung Nguyen, Jongyoon Han

**Affiliations:** 1 School of Mechanical and Aerospace Engineering, Nanyang Technological University, 50 Nanyang Avenue, Singapore; 2 BioSystems and Micromechanics (BioSyM) IRG, Singapore-MIT Alliance for Research and Technology (SMART) Centre, 1 Create Way, #03 Enterprise Wing, Singapore; 3 School of Biological Sciences, Nanyang Technological University, 60 Nanyang Drive, Singapore; 4 Infectious Diseases IRG (ID), Singapore-MIT Alliance for Research and Technology (SMART) Centre, 1 Create Way, #03 Enterprise Wing, Singapore; 5 Lee Kong Chian School of Medicine, Nanyang Technological University, 11 Mandalay Road, Singapore; 6 Queensland Micro- and Nanotechnology Centre, Griffith University, 170 Kessels Road, QLD 4111, Australia; 7 Department of Electrical Engineering & Computer Science, Massachusetts Institute of Technology, Room 36-841, 77 Massachusetts Avenue, Cambridge, MA, USA; 8 Department of Biological Engineering, Massachusetts Institute of Technology, Cambridge, MA, USA

## Abstract

Despite significant advancements over the years, there remains an urgent need for low cost diagnostic approaches that allow for rapid, reliable and sensitive detection of malaria parasites in clinical samples. Our previous work has shown that magnetic resonance relaxometry (MRR) is a potentially highly sensitive tool for malaria diagnosis. A key challenge for making MRR based malaria diagnostics suitable for clinical testing is the fact that MRR baseline fluctuation exists between individuals, making it difficult to detect low level parasitemia. To overcome this problem, it is important to establish the MRR baseline of each individual while having the ability to reliably determine any changes that are caused by the infection of malaria parasite. Here we show that an approach that combines the use of microfluidic cell enrichment with a saponin lysis before MRR detection can overcome these challenges and provide the basis for a highly sensitive and reliable diagnostic approach of malaria parasites. Importantly, as little as 0.0005% of ring stage parasites can be detected reliably, making this ideally suited for the detection of malaria parasites in peripheral blood obtained from patients. The approaches used here are envisaged to provide a new malaria diagnosis solution in the near future.

Malaria is a mosquito-borne infectious disease that affects 3.4 billion people - about half of the world’s population^1^. According to the malaria report 2013, there were an estimated 207 million cases of malaria infections in year 2012, causing a toll of 627,000 deaths^2^. The disease is most prevalent in tropical and subtropical countries of Africa, South America and South-East Asia. Obligate intracellular parasites of the genus *Plasmodium* are the cause of malaria. During its complex life cycle the parasite infects red blood cells (erythrocytes) and replicates within. The parasite directly ingests hemoglobin from the infected erythrocyte as a source of essential metabolites and energy^3^. While there are more than 120 species of *Plasmodium*, only 5 species are known to infect humans; *P. falciparum*, *P. vivax*, *P. ovale*, *P. malariae*, and *P. knowlesi*. *P. falciparum* is the most common, and is responsible for more than 90% of malaria death^1^. During the maturation in the erythrocyte, three distinct morphological stages can be observed microscopically - the ring, trophozoite, and schizont stage. The cyclical invasion and reinvasion of malaria parasites in red blood cells causes the number of infected red blood cells to increase exponentially, resulting in the symptoms such as fever, headache, rigors, and nausea usually associated with malaria.

The major challenges in current clinical malaria diagnostics are in obtaining a sensitive, robust, fast, and inexpensive measurement from patients’ blood samples. One of the leading causes of the high mortality rate in malaria is the delay in medical diagnosis and treatment^4^. Prompt diagnosis and timely treatment for malaria is often difficult to come by in poverty-stricken countries with limited medical resources, while developed countries, with very few cases, often lack experience and expertise in malaria diagnosis^5^. Most malaria deaths can be prevented if diagnosis and effective treatment were administered within 24 hour after the onset of first symptom^6^. Physicians need the information on the severity of infection, often expressed in parasitemia (the ratio of infected to the total number of red blood cells) to determine the treatment schedule for malaria patients. Furthermore, anti-malarial drugs, such as artemisinin - the current last line of defense against malaria, have become less effective in malaria treatment as malaria parasite have adapted and developed resistance to the drugs^7^,^8^.

Giemsa-stained microscopic examination of thick blood smear has remained the gold standard for laboratory diagnosis of malaria for more than a century^5^,^9^. The procedure is labor intensive, time consuming and requires well trained and highly experienced personnel. In recent years, rapid diagnostic tests (RDT) such as the dipsticks were developed to aid malaria detection in malaria endemic area. However, negative RDT test results cannot be accepted directly without confirmation by microscopic examination^10^,^11^. The polymerase chain reaction (PCR) method has a high detection sensitivity of 0.0001% or as few as five parasites μL^−1^ of blood^11^,^12^. However, the process takes up to six hour per sample and is costly to perform^13^. Factors that preclude PCR as a suitable method for diagnosis tool in rural areas or in routine clinical setting include limited financial resources, inadequate infrastructures, and lack of well-trained technicians^5^,^12^. So, even to date, access to fast, sensitive and reliable diagnostic tool is still lacking.

The miniaturization of magnetic resonance relaxometry (MRR) systems has been attracting considerable interest in recent years due to the promising applications in disease biomarker detection, point-of-care diagnosis, and cancer screening by measuring the transverse relaxation rate, *R*_*2*_ of proton present in the bio-samples^14^,^15^,^16^,^17^. There are two main benefits to the miniaturization of MRR systems; it offers higher mass sensitivity and requires smaller sample volume than conventional nuclear magnetic resonance systems, which require high-strength, spatially uniform magnetic field^18^,^19^. Furthermore, each *R*_*2*_ measurement only takes approximately 1 minute. Recently, we have demonstrated that the presence of paramagnetic malaria pigment or hemozoin crystallites in malaria infected red blood cells (*i*RBCs) serves as the natural biomarker for malaria detection^20^. The bulk magnetic susceptibility of the *i*RBCs (Table 1) is significantly higher than the healthy red blood cells (*h*RBCs), which in turn induces a substantial change in the transverse relaxation rate. Nonetheless, MRR detection has a major drawback in that it measures the absolute value of a blood sample instead of detecting a relative change in the *R*_*2*_ value.

On the other hand, the separation of malaria *i*RBCs was made possible with the development of lab on a chip (LOC) or micro total analysis systems (μTAS). Gascoyne *et al*. exploited the difference in the dielectric properties of *i*RBCs and *h*RBCs to perform dielectrophoretic (DEP) separation in a microfluidic chamber^21^. While the technique has the ability to trap *i*RBCs at a localized position, the application of relatively high electric field causes Joule heating and could dramatically increase the temperature of the suspending buffer^22^, and affect the erythrocytes for downstream measurements. Ribaut *et al*.^23^ and Zimmerman *et al*.^24^ concentrate and purify *i*RBCs through magnetic columns (MACS) and magnetic deposition microscopy (MDM), respectively. However, both techniques have a serious limitation in that they were unable to separate ring stage *i*RBCs, which is important for the human *P. falciparum* malaria diagnosis. Previously, we have presented a deformability based separation method known as margination, where the stiffer malaria *i*RBCs are segregated towards the sidewalls of microchannel^25^. In contrast to the DEP or MDM methods, microfluidic margination offers the convenience of not needing external electrical and magnetic fields for *i*RBCs separation.

In this work, we develop a malaria diagnosis protocol that combines the use of microfluidics for the separation of *i*RBCs and MRR system for the rapid, label-free detection of malaria parasites. We mitigate the *R*_*2*_ baseline restriction by separating the *i*RBCs from the *h*RBCs through margination and compare the relative change in the relaxation rates of the separated blood samples. The diagnostic method that we propose here has similar detection sensitivity to PCR based methods and, at the same time, is one order of magnitude more sensitive than the standard Giemsa microscopic method. We improved the throughput of the microfluidic margination device by employing a multiplex margination design with 7 parallel margination channels, and demonstrated that the device could effectively separate the *h*RBCs from *i*RBCs of all infection stages (ring, trophozoite, and schizont). Due to the margination effect, the stiffer *i*RBCs are segregated towards the side outlet of the microfluidic channel, while the blood sample collected at the middle outlet is depleted of *i*RBCs. Thus, the marginated blood sample collected at the middle outlet has a lower *R*_*2*_ value compared to the inlet. The ability to perform relative *R*_*2*_ measurements alleviated the need for establishing a universal *R*_*2*_ baseline for MRR malaria diagnosis.

## Results and discussion

### Characterization of margination efficiency

**Figure 1:**
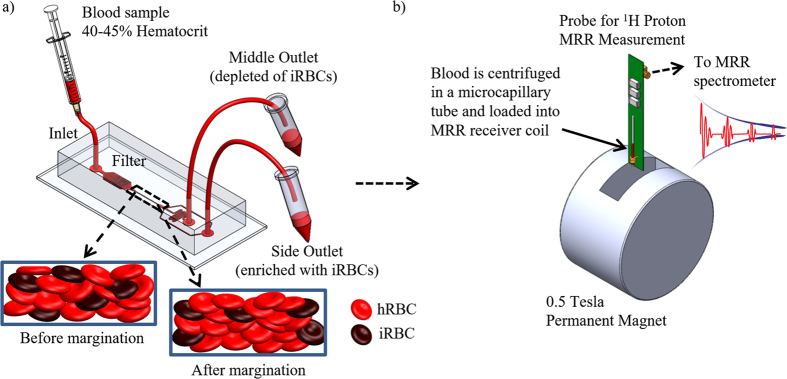
Schematic diagrams. (**a**) Schematic diagram of the margination device. The microfluidic separation device that has one inlet, two outlets, and a margination channel of 50 μm in width and 20 mm in length. The insets show the schematic of the cross-sectional view of *h*RBCs and *i*RBCs distribution before and after passing through the margination channel. The initially randomly distributed stiffer *i*RBCs are marginated towards the sidewall, while the more deformable *h*RBCs migrate towards the axial center of the microchannel. (**b**) Schematic diagram of the bench top MRR system for *R*_*2*_ measurement. The blood sample is centrifuged in a microcapillary tube and loaded into MRR receiver coil placed inside a 0.5 T permanent magnet. The receiver coil is connected to a bench-top spectrometer for MRR measurements.

**Figure 2:**
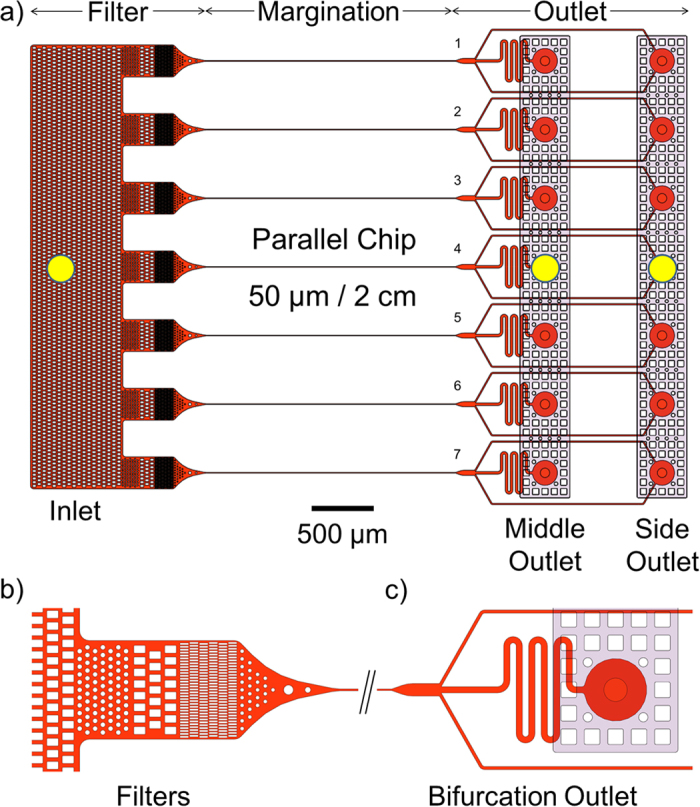
Channel Design. (**a**) The margination channel design. The microfluidic margination device comprises of two microfluidics components: a margination layer and a cover layer. The margination layer consists of seven parallel margination channels, each with a width of 50 μm, a thickness of 10 μm, and a length of 2 cm. The yellow circles indicate the tubing ports’ location for the inlet, middle, and side outlets of the margination device. (**b**) Zoom in of the inlet and filtration unit. The microstructures and pillars effectively trap the coagulated blood and large debris to avoid channel blockage in the long and narrow margination channels. (**c**) Bifurcation outlet at the end of each margination channel. The *h*RBCs migrates to the axial axis of the microchannel and exit via the middle outlet, while the stiffer *i*RBCs are marginated to the sidewalls and exit via the side outlets.

**Figure 3:**
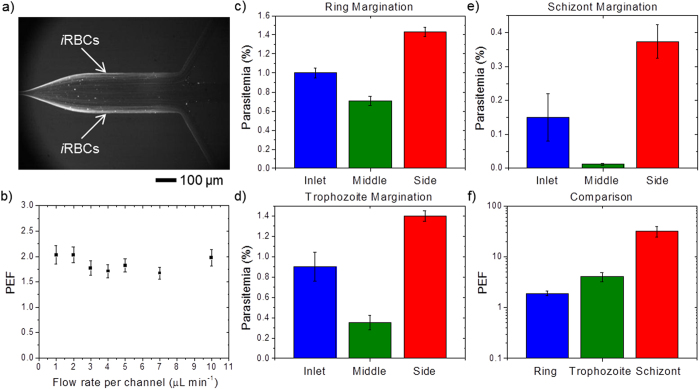
Characterization of margination efficiency. (**a**) DAPI fluorescence imaging at the bifurcation exit. The fluorescently labeled *i*RBCs with 5% parasitemia level (~20% ring, ~80% schizont) are marginated effectively toward the sidewalls of the microchannel. (**b**) The parasitemia enhancement factor, *PEF* at various flow rates for 1% ring *i*RBCs. Increasing the flow rate per channel from 1–10 μL min^−1^ does not affect the *PEF* substantially. Therefore, the flow rate could be increased to improve the throughput of the margination system. (**c**)–(**e**) Margination results for (**c**) ring, (**d**) trophozoite, and (**e**) schizont. The parasitemia levels of the *i*RBCs at the side outlets are significantly higher than the middle outlets for all infection stages. In other words, the middle outlet is depleted of *i*RBCs, while the side outlet is enriched with *i*RBCs. (**f**) Comparison of parasitemia enrichment factor for ring, trophozoite and schizont. As the stiffness of *i*RBCs increases exponentially as the infection stage progresses from ring to trophozoite and schizont, the margination efficiency also increases exponentially, 1.9 ± 0.2, 4.1 ± 0.8, and 32.1 ± 7.6, for ring, trophozoite, and schizont, respectively.

The efficient enrichment of *i*RBC would be of significant benefit for the subsequent diagnostic of malaria, particularly in cases of very low parasitemia levels. In our previous work^25^, we demonstrated the margination of malaria *i*RBCs with a single margination channel. To improve this further, we evaluated the margination efficiency of the multiplex margination design (Fig. 1(a) and Fig. 2) with 7 parallel margination channels. With the margination effect, the stiffer *i*RBCs are segregated towards the sidewall, while more deformable *h*RBCs migrate towards the axial center of the microchannel. Figure 3(a) shows the DAPI fluorescence imaging at one of the bifurcation exits at the end of a margination channel with a total flow rate of 14 μL min^−1^, or 2 μL min^−1^ per channel. As the margination channel width-height ratio is 5:1, the channel height of 10 μm is taken as the characteristic length for the Reynolds number, *Re* estimation. Hence, assuming the whole blood density as 1056 kg m^−3^ and viscosity^26^ as 3.5×10^−3^ Pa s, the *Re* for flow rate of 2 μL min^−1^ is approximately 0.2. For all experiments, the hematocrit of the blood sample at the inlet is kept at 45–50%. We can clearly observe that the fluorescently labeled *i*RBCs with ~5% parasitemia level (~20% ring, ~80% schizont) are marginated effectively toward the sidewalls of the microchannel.

Since clinical samples contain only circulating ring stage parasites, we further evaluated the margination efficiency to separate ~1% ring *i*RBCs at various margination flow rates. With the help of a fluorescence activated cell sorting (FACS) flow cytometer, we could characterize the margination efficiency by estimating the parasitemia level of the blood collected from the middle and side outlets accurately. Figure 3(b) shows the parasitemia enrichment factor, *PEF*, for single channel flow rate of 1–10 μL min^−1^ (Re ≈ 0.1–1) for ~1% ring *i*RBCs. The parasitemia enrichment factor, *PEF* is defined as the parasitemia ratio between the side and middle outlet. Thus, a higher *PEF* value would indicate a higher margination sorting efficiency. As the *PEF* value remains relatively constant throughout the flow rate range, we can infer that increasing the margination flow rate from 1 to 10 μL min^−1^ does not adversely affect the margination efficiency substantially. Therefore, the flow rate could be increased to improve the throughput of the margination system without compromising the margination performance. With multiplex margination, throughput of the microfluidic separation step is improved to a total flow rate of up to 70 μL min^−1^, which reduces the margination process time to approximately 15 minutes.

Figure 3(c–e) show the margination results for ring, trophozoite, and schizont, respectively. With the margination effects (see Methods), the parasitemia levels of the *i*RBCs at the side outlets are higher than the middle outlets for all three infection stages. In other words, the middle outlet is depleted of *i*RBCs, while the side outlet is enriched with *i*RBCs. The comparison of parasitemia enrichment factor for ring, trophozoite and schizont is summarized in Fig. 3(f). Since the stiffness of *i*RBCs increases exponentially as the infection stage progresses from ring to trophozoite and schizont (Table 1)^27^, the margination efficiency also increases accordingly. The multiplex margination device could separate *i*RBCs for all three stages of infection - ring, trophozoite, and schizont effectively from *h*RBCs with a parasitemia enrichment factor and standard deviation of 1.9 ± 0.2, 4.1 ± 0.8, and 32.1 ± 7.6, respectively.

### Establishing healthy baseline

**Figure 4:**
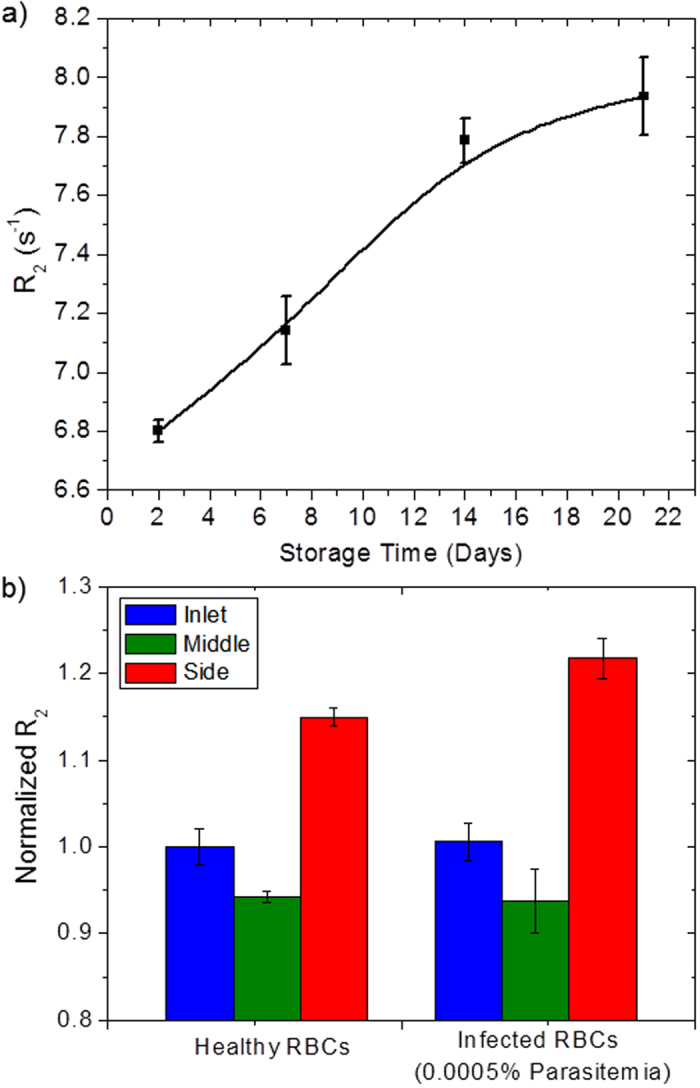
RBC *R*_2_ measurements. (**a**) Weekly *R*_*2*_ measurement of a healthy blood sample for up to three weeks of post storage. As the *h*RBCs age, the *R*_*2*_ value increased as much as 16.7% from day two to day 21. (**b**) The normalized spin-down *R*_*2*_ values of *h*RBCs and *i*RBCs for the blood samples collected at inlet, middle, and side outlets. However, for *h*RBCs, the side outlet *R*_*2*_ value is significantly higher than the inlet and middle *R*_*2*_. For healthy blood sample, the margination device also separates the stiffer old RBCs from the more flexible young RBCs. When the *h*RBCs are spiked with the presence of 0.0005% *i*RBCs, the *R*_*2*_ value of the side outlet increases further since the *i*RBCs are marginated towards the sidewalls.

Recently, we have shown that micro magnetic resonance relaxometry can be used as a new approach for malaria diagnostics^20^. The malaria infected *i*RBCs have higher transverse relaxation rate, *R*_*2*_ than the healthy *h*RBCs due to the presence of paramagnetic hemozoin crystallites in the infected blood^20^. These paramagnetic crystallites increase the bulk magnetic susceptibility of the *i*RBCs and induce a considerable increase in the *R*_*2*_ relaxation rate. Hence, the detection of the subtle increase in the *R*_*2*_ due to the presence of malaria parasites forms the basis of MRR malaria detection. However, MRR detection method measures the absolute *R*_*2*_ value of the blood sample instead of a relative change in the *R*_*2*_ value. The healthy absolute *R*_*2*_ value varies from individual to individual depending on numerous factors such as age, diet, genetic variation, and hematocrit number, and methemoglobin content. Furthermore, the *R*_*2*_ value of *h*RBCs increases with the number of post storage days when stored under hypothermic condition at 4 °C (Fig. 4(a)). The *R*_*2*_ value of that healthy blood sample increased from 6.80 ± 0.04 s^−1^ on day two to 7.94 ± 0.13 s^−1^ on day 21, an increment of 16.7%. Since the *R*_*2*_ value of the *h*RBCs increases as the RBCs age, establishing a universal healthy baseline *R*_*2*_ value for all individuals is rather impractical. Therefore, the malaria detection method based on the measurement of absolute *R*_*2*_ value needs to overcome the problem of the variation in the healthy baseline to be fully effective.

In order to overcome the *R*_*2*_ baseline challenge, we separated *i*RBCs from the *h*RBCs through microfluidic margination and compared the relative change in the relaxation rates, *ΔR*_*2*_ of the separated blood samples. Figure 4(b) shows the normalized *R*_*2*_ values of *h*RBCs and *i*RBCs for the marginated blood samples collected at inlet, middle, and side outlets, where the absolute *R*_*2*_ values of each sample are divided with the absolute *R*_*2*_ value of the inlet *h*RBCs. The blood collected at the inlet refers to the blood sample obtained by remixing the bloods collected from the middle and side outlets after passing through the microfluidic channel, thus ensuring a fair *R*_*2*_ comparison between the inlet with middle and side outlets. The *R*_*2*_ relaxation rates of the collected blood samples are measured using the spin-down method described in the sample preparation section (see Methods). Interestingly, for *h*RBCs, the side outlet *R*_*2*_ value is approximately 15% higher than the inlet, while the middle *R*_*2*_ is approximately 6% lower than the inlet *R*_*2*_. A similar trend is observed for *i*RBCs. When the *h*RBCs are spiked with 0.0005% *i*RBCs, the *R*_*2*_ value of the side outlet increased further by approximately 7% since the *i*RBCs are marginated towards the sidewalls.

The observed phenomenon, that the side outlet *R*_*2*_ value is always significantly higher than the inlet, while the middle *R*_*2*_ is lower than the inlet *R*_*2*_ for *h*RBCs, may indicate that the margination device also separates stiffer and older RBCs from more flexible, younger RBCs. As the average life span of RBCs *in vivo* is about 120 days^28^, the *h*RBCs circulating in the human blood stream is made up of a distribution of RBCs of various age and deformability. The stiffness of RBC increases substantially during the aging process, and is up to 134% less deformable on day 36^29^. RBCs are naturally damaged by oxidation during aging^30^, and cross-links are formed between the hemoglobin (Hb) and membrane proteins in the RBC^31^. Aging of RBC is associated with numerous changes, which include a reduction of intracellular glutathione (GSH) predisposing the cell to oxidative damage^32^. With diminished reducing activity, and a constant auto-oxidation rate of Hb, there is gradual accumulation of methemoglobin (metHb), a ferric (Fe^3+^) derivative of Hb, in the senescent RBC^33^. MetHb can be further oxidized to a ferryl (Fe^4+^) state under constant oxidative stress^34^. Such oxidative stress ultimately leads to Hb denaturation, band 3 clustering, and autologous immunoglobulin G (IgG) binding and general stiffening which marks the RBC for splenic clearance^32^,^35^. RBCs in the ferric and ferryl states are paramagnetic, and thus are responsible for the higher *R*_*2*_ relaxation rates of the marginated stiff aged RBCs collected at the side outlet.

**Figure 5:**
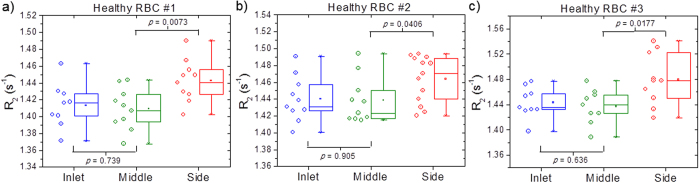
Establishing healthy baseline with the saponin lysis method. The *R*_*2*_ values at the inlet, middle, side for three different healthy individuals *h*RBCs using the lysis method. The box plots show the lower quartile, median, and upper quartile of their respective sample distributions. While the side *R*_*2*_ values remains significantly higher than the middle, the middle *R*_*2*_ values are non-distinguishable statistically from the inlet *R*_*2*_ with *P*-value of 0.739, 0.905, and 0.636, respectively.

Due to the margination of aged RBCs, there exists a substantial relative change in the relaxation rates, *ΔR*_*2*_ of the separated blood samples even for *h*RBCs (Fig. 4b). To overcome this challenge we lyse the RBC with saponin after margination but prior to the MRR measurement as this diminishes the relative difference in *R*_*2*_ relaxation rates between the blood samples collected at the inlet and middle, enabling us to establish a healthy baseline necessary for reliable diagnosis. Figure 5 shows the *R*_*2*_ values at the inlet, middle, side for three different healthy individuals using the saponin lysis method (see Methods). The box plots show the lower quartile, median, and upper quartile of their respective sample distributions. For all three cases, we observed that, while the side *R*_*2*_ values remains significantly higher than the middle, the middle *R*_*2*_ values has become statistically non-distinguishable from the inlet *R*_*2*_ (two-tailed paired student *t*-test (*n* ≈ 10) *P*-values of 0.739, 0.905, and 0.636, respectively). Due to the variation in *R*_*2*_ values between measurements, it is necessary to take 5–10 measurements per sample to prevent false positives and false negatives, thus the MRR measurement time per sample is approximately 5–10 minutes. Lysing the *i*RBCs with saponin releases intact *P. falciparum* parasites from the membrane of erythrocytes and it would therefore be expected that these intact parasites would remain to provide a detectable increase in the *R*_*2*_ values during MRR measurements. If this is indeed correct, we expect that as *i*RBCs are marginated towards the sidewalls, the middle outlet is depleted of *i*RBCs. Hence, the middle *R*_*2*_ values would be expected to be significantly lower compared to the inlet while it would be statistically non-distinguishable if the sample contains only *h*RBCs.

### Validation of the lysis method for reliable parasite detection

**Figure 6:**
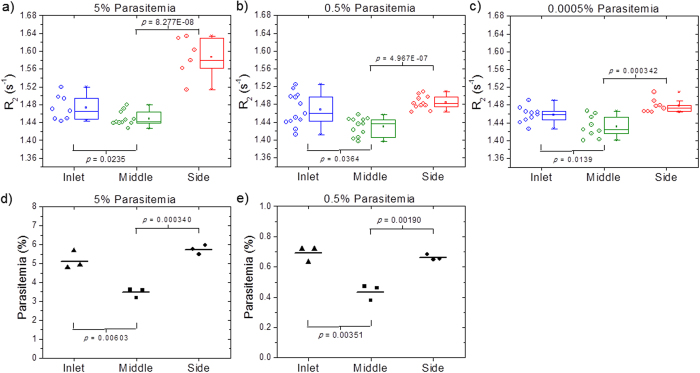
Dilution test. (**a**)–(**c**) The *i*RBCs of ~10% parasitemia obtained from malaria culture is diluted to 5%, 0.5%, and subsequently 0.0005% parasitemia. The *i*RBCs composition consists of 37% ring and 63% schizont. The box plots show the lower quartile, median, and upper quartile of their respective sample distributions. As expected, the lysed *R*_*2*_ values of the middle is significantly lower than the inlet with *t*-test *P*-value < 0.05, while the side *R*_*2*_ is significantly higher than the middle; (**d**)–(**e**) FACS parasitemia estimation for the 5% and 0.5% samples. As the middle outlet is depleted of *i*RBCs, the parasitemia level at the middle outlet is significantly lower than the inlet and side outlet. The result for 0.0005% parasitemia sample is not available since it is beyond the detection limit of FACS.

To establish whether the comparison of inlet vs middle outlet in combination with saponin lysis was able to reliably detect low level of parasites, we evaluated this approach using samples with different parasitemia. Figure 6(a–c) shows the *R*_*2*_ values of blood samples collected at inlet, middle and side for an *i*RBCs sample at parasitemia levels of 5%, 0.5%, and 0.0005%. All these samples contained a mixed infection of 37% ring and 63% schizont. The box plots show the lower quartile, median, and upper quartile of their respective sample distributions. For all three parasitemia levels, the *R*_*2*_ values of the middle is significantly lower than the inlet (*t*-test *P*-value < 0.05), while the side *R*_*2*_ is significantly higher than the middle. This confirms that the depletion of *i*RBC in the middle outlet can be directly measured using the saponin lysis approach even at parasitemia levels as low as 0.0005%, providing us with an important tool for malaria parasite detection. In contrast as shown in Fig. 6(d,e) FACS analysis of the different samples after margination but before saponin lysis is only able to provide data for the 5% and 0.5% sample as the 0.0005% sample is beyond its detection limit. The FACS data shows that as the *i*RBCs are marginated towards the side outlet, and although the margination process does not remove all existing *i*RBCs from the middle channel (as shown in Fig. 6(d,e)), the parasitemia level at the middle outlet is significantly lower than both the inlet and side outlet.

### Limit of detection for ring stage parasites

**Figure 7:**
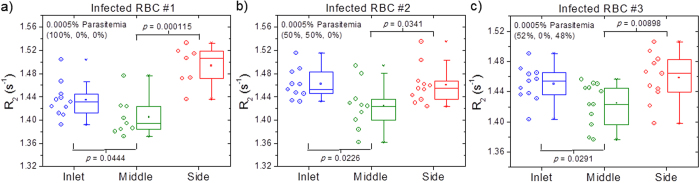
Limit of detection. The *R*_*2*_ values at the inlet, middle, side for three different *i*RBCs with 0.0005% parasitemia with the lysis method. The parentheses indicate the percentage composition of each malaria infection stage (ring%, trophozoite%, and schizont%). As the *i*RBCs are marginated towards the sidewalls, the middle outlet is depleted of *i*RBCs. Hence, the middle *R*_*2*_ values would be significantly lower (with *P*-value < 0.05) compared to the inlet with the presence of *i*RBCs. For the sample of 100% ring at 0.0005% parasitemia in (**a**), the *P*-value is 0.0444 which indicate that the *R*_*2*_ distributions of the middle is statistically distinguishable from the inlet. The presence of middle and late stage infection of trophozoite and schizont in (**b**) and (**c**) increases the *P*-value further to 0.0226 and 0.0291, respectively.

For any diagnostic approach it is crucial that it can detect solely circulating young ring stages as later stage *i*RBCs, trophozoites and schizonts, are usually sequestered in the deep tissues, leaving only early stage ring *i*RBCs in the human peripheral blood. We therefore evaluated the limit of detection using samples containing different proportions of ring (R), trophozoite (T), and schizont (S) stage parasites. Three samples at extremely low parasitemia level of 0.0005% parasitemia with a composition estimated by Giemsa microscopic observation and FACS of (a) (100% R, 0% T, 0% S); (b) (50% R, 50% T, 0% S); (c) (52% R, 0% T, 48% S) were tested. Fig. 7 shows the *R*_*2*_ values at the inlet, middle, and side for the three samples of *i*RBCs. In all three cases, the *R*_*2*_ relaxation rates of the sample collected at the middle outlet are significantly lower (*P*-value < 0.05) compared to the inlet. As seen in Fig. 7(a), even for the 100% ring sample, statistically significant (*P*-value is 0.0444) differences between the inlet and middle outlet sample can be detected. This confirms that the malaria diagnostic technique that combines the use of microfluidic separation and MRR system is able to detect early malaria infection at the parasitemia level of 0.0005%, without prior determination of patient-specific healthy baseline.

## Conclusions

We have successfully developed a malaria diagnosis technique that combines the use of microfluidics for the separation of *i*RBCs and MRR system for a rapid, label-free detection of malaria parasites. The *i*RBCs are separated from the *h*RBCs through margination - a naturally occurring hemodynamic phenomenon where more deformable *h*RBCs experience a lateral migration towards the axial centre of the microchannel, displacing stiffer *i*RBCs to the sidewalls of the margination channel. Crucially we have demonstrated that this approach is capable of reliably detecting ring stage parasites at parasitemia as low as 0.0005%. In the near future, a Lab-on-a-Chip solution with MRR functionality integrated is envisaged for malaria diagnosis in the clinical setting. Both margination-based microfluidic separation system and MRR detection system have a room for further optimization, which will bring about more sensitive diagnostics, and, potentially, earlier detection of malaria cases. While the cost of the miniaturized MRR system maybe still expensive (several thousand US dollar) for developing nations, the cost per assay (<50 cents) is affordable. Pushing the detection sensitivity of malaria parasite to the lowest level possible would have significant implication in the clinical management of malaria in endemic areas, especially in the light of the possibility of latent, extremely low level parasitemia in non-symptomatic populations.

## Methods

### Cell margination is a naturally occurring hemodynamic phenomenon^36^ in our microcirculatory system

Stiffer nucleated white blood cells (leukocytes) are segregated towards the vessel wall to initiate leukocyte rolling, adhesion and extravasation at the blood vessel wall’s endothelia cells of the injury inflammation site^37^. The same margination principle could also be applied to displace stiffer malaria *i*RBCs towards the sidewalls (inset of Fig. 1(a))^25^. The margination effect is primarily attributed to RBCs axial migration resulting from wall induced migration and shear-induced diffusion^38^. In a wall-bound Poiseuille shear flow, deformable RBCs undergo change in shape and experience an inwardly directed wall induced pressure gradient due to the velocity difference in the laminae of the sheared suspension^25^,^39^,^40^. These wall-induced migration pressure gradient tend to push the RBCs away from the channel wall towards the axial center of the microfluidic channel, leaving a cell depleted plasma layer near the channel wall - the Fahraeus effect^38^,^41^,^42^. The stiffer *i*RBCs are more spherical in shape and experience smaller wall induced lift velocities than the *hRBCs*^41^. On the other hand, the shear induced diffusion due to the hydrodynamic interactions and mechanical collisions between the RBCs in the shear flow displace the stiffer malaria *i*RBCs towards the sidewall^41^.

While *h*RBCs are highly deformable, the *i*RBCs stiffen upon invasion of the large non-deformable intracellular parasite, and changes its morphological, mechanical, and electrical properties^43^,^44^. Furthermore, the parasite releases proteins that trigger the cross-linking of the spectrin network in the *i*RBC’s phospholipid bilayer membrane, thus increases the rigidity of the *i*RBCs^45^. The elastic modulus of ring, trophozoite and schizont are approximately 2, 2.7 and 6.7 times higher than the *h*RBC, respectively (Table 1)^27^. Therefore, through the biomimetic margination microfluidic separation device, we could effectively segregate the stiffer *i*RBCs towards the side outlet of the channel, while the blood sample collected at the middle outlet is depleted of *i*RBCs (Fig. 1(a)). Subsequently, we perform the relative *R*_*2*_ measurements with a bench-top 0.5 T MRR system (Fig. 1(b)).

### Channel design

The microfluidic margination device comprises of two microfluidic components: a margination layer and a cover layer. Due to the narrow cross-section and relatively long nature of the margination channel, the fluidic resistance for the margination channel is high. Thus, the maximum achievable flow rate is limited, and subsequently limits the ability for high throughput margination. In order to increase the throughput and reduce the processing time of the microfluidic separation step, we opt to perform multiplex margination. The margination layer consists of seven parallel margination channels, each with a width of 50 μm, a thickness of 10 μm, and a length of 2 cm. Figure 2(a) shows the schematic diagram for the design of the microfluidics margination device. At the end of each margination channel, the blood streams are separated into a bifurcation channel configured in a 1:2:1 manner (Fig. 2(c)). The *h*RBCs tend to move to the axial center of the microfluidic channel and exit through the middle outlet, while the stiffer *i*RBCs are segregated to the channel sidewalls and exits through the side outlets. In addition, we have also implemented multiple microstructures and pillars with gaps ranging from 25 μm to 200 μm. Figure 2(b) shows the magnified view of the filtration units at the inlet. These microstructures and pillars act as the filtration unit at the inlet to effectively trap any coagulated blood or debris that might block the narrow margination channel should they be allowed to pass through the microchannel. The cover layer consists of two large microchannels (transparent rectangular channels shown in Fig. 2(a) and is then aligned and bonded on top of the margination layer. The purpose of cover layer is to eliminate the need of connecting 14 tubings for blood collection at the outlets. The bloods from the middle and side outlets are pooled and exits via two large microchannels of the cover layer. The yellow circles indicate the tubing ports’ location for the inlet, middle, and side outlets of the margination device.

### Device fabrication

The microfluidic margination device was fabricated in polydimethylsiloxane (PDMS) using standard photolithography technique. An approximately 10 μm thick SU8-3005 (Micro-Chem Inc., USA) negative photoresist was spin-coated onto a 4” silicon wafers at 500 rpm for 10 s with an acceleration of 100 rpm s^−1^, followed by 750 rpm for 30 s with an acceleration of 300 rpm s^−1^. The substrate was then soft-baked at 65 °C for 1 minutes and 95 °C for 3 minutes. UV exposure was performed with a mask aligner (MJB-4, SussMicrotec, Germany) at 200 mJ cm^−2^ for 16 s. Subsequently, post exposure bake is carried out at 65 °C for 1 minutes and 95 °C for 2 minutes. The substrate is then developed for 2 minutes. PDMS with a 10:1 mixture ratio for base and curing agent (Sylgard 184, Dow Corning Inc., USA) was casted onto the SU8 master and cured at 80 °C for 8 hours. Inlet and outlets ports were punched with a 0.75 mm PDMS puncher (Harris-Unicore Inc., USA) on the cured PDMS device. Finally, the PDMS device was cleaned in an ultrasonic cleaner with ethanol and DI-water for 15 minutes, respectively. The entire process was repeated to fabricate the PDMS chip that contains the cover layer.

After fabricating the PDMS chips for both the margination layer and cover layer, the next step was to assemble the multilayer microfluidic separation device. First, the microfluidics chip that contain the margination layer was bonded onto a pre-cleaned microscope glass slide (75 mm × 50 mm, Corning Inc., USA) with a plasma bonder (Covance, Femto Science Inc., South Korea) for 90 s. Second, the PDMS chip that contains the cover layer was then visually aligned and bonded onto the top surface of the PDMS chip containing the margination layer. The assembled microfluidic device was then kept in an oven at 80 °C for 8 hours to improve the PDMS bonding strength. Lastly, a tubing with a G23 needle end was inserted into the inlet and sealed with epoxy glue (Selleys Araldite Inc., Australia) to enhance the sealing.

### Malaria culture

Healthy non-malarial immune adult volunteer blood donors were recruited, and brought to the National University Hospital of Singapore Blood Bank for phlebotomy services. The protocol was approved by the Institutional Review Board of National University of Singapore (NUS-IRB 10-285). Informed consent was taken from all volunteers, and the methods were carried out in accordance with the approved protocol and guidelines. Whole venous blood was collected in Citrate-Phosphate-Dextrose-Adenine-1 (CPDA-1, JMS). The white blood cells (leukocyte) were removed via centrifugation and the removal of the buffy coat layer. Malaria parasites, *P. falciparum* strain 3D7 (MR4, USA) was cultured at 2.5% haematocrit using human erythrocytes in malaria culture media (10.43 g RPMI 1640 powder (Gibco, USA), 25 mL 1 M HEPES (Gibco, USA), 2 g NaHCO_3_ (Sigma-Aldrich, USA), 5 g AlbuMAX® (Gibco, USA), 0.05 g of hypoxanthine (Sigma-Aldrich, USA) in 1 mL of 1 M NaOH, and 25 mg gentamicin (Gibco, USA) in 1 L milli-Q water)^46^. Cultures were kept in a Heracell 150 incubator (Thermo Scientific, USA) at 37 °C in an atmosphere of 5% CO_2_, 3% O_2_, and 92% N_2_. Ring stage parasites were synchronized using 2.5% D-sorbitol, while schizont stage parasites were purified using differential Percoll gradient centrifugation^46^. The parasitemia of the parasite culture was quantified either by standard Giemsa-staining of blood smear or by flow cytometry. For flow cytometry analysis, infected erythrocytes were fixed in 4% formaldehyde, followed by permeabilization with 0.5% v/v saponin solution in flow buffer (0.2% bovine serum albumin (BSA), 0.05% sodium azide in 1 × phosphate buffer solution (PBS)). Thereafter, parasite’s DNA was stained using Hoechst 33342 at a concentration of 20 μg mL^−1^ (Thermo Scientific, USA).

### Sample Preparation

When the parasitemia of the *i*RBCs reaches ~10%, the infected blood sample is washed with 1 × PBS three times to remove the culture media and resuspended in 1 × PBS at 10% hematocrit. Subsequently, we prepare an infected blood sample at 5% parasitemia by spiking the corresponding amount of *i*RBCs into *h*RBCs from a healthy donor. The 5% parasitemia blood sample is then serially diluted with *h*RBCs to obtain blood samples with lower parasitemia of 0.5%, 0.05%, 0.005%, and 0.0005%. The hematocrit of the blood samples are then adjusted to 45–50% for the microfluidic margination process. The *R*_*2*_ relaxation rates of the blood samples can be measured by two methods: (a) “spin-down”, and (b) “lysis” methods. In the “spin-down” method, the samples are loaded into heparinized microcapillary tubes (22–260–950, Fisher Scientific, USA) via capillary forces. The microcapillary tubes are then sealed with a vinyl-plastic compound (Critoseal, Krackeler Scientific,USA) and centrifuged (Sorvall Legend Micro 21, Thermo Scientific, USA) at 13,000 g for one minutes. A total spun down cell volume of 4 μL is required for each MRR measurement. On the other hand, the procedure for the “lysis” method is similar to the “spindown” method except that the blood cells are lysed before being transferred into the microcapillary tubes for MRR measurements. First, the total volume of blood cells suspended in 1 × PBS required for obtaining a spun down cell volume of 4 μL is determined. Subsequently, the blood sample is centrifuged at 3,000 g for one minute. A portion of the supernatant of the sample is removed such that the total sample volume is 15 μL. The blood sample is then lysed with 5 μL of 1% v/v saponin lysis buffer (Sigma-Aldrich, USA).

### Experiment setup

Prior to the experiment, the microfluidic margination device is primed with 1 × PBS supplemented with 0.5% v/v BSA. The blood sample is filled in a 1 mL glass syringe (Hamilton Inc., USA) and pumped into the margination device with a syringe pump (Fusion 400, Chemyx Inc., USA) at total flow rates ranging from 7 μL min^−1^ to 70 μL min^−1^ with flexible Tygon© tubing (0.02” × 0.06”, 06419-01, Cole-Parmer Inc., USA). The flow of the *i*RBCs and *h*RBCs are observed under an inverted epi-fluorescence microscope (Olympus IX71, Olympus Inc., Japan) equipped with a high speed CCD camera (Phantom v9, Vision Research Inc., USA). The marginated blood samples are then collected from the middle and side outlets in 1.5 mL microcentrifuge tubes. We evaluated the performance of the margination separation device by running FACS analysis with a BD™ LSR II flow cytometer (BD Biosciences, USA) on the collected blood samples. The data from the cytometer is then analyzed with FACS Diva (BD Biosciences, USA) and FlowJo (TreeStar Inc., USA).

The proton ^1^H transverse relaxation rate, *R*_2_ of the blood samples from the middle and side outlets are measured with a bench-top nuclear magnetic resonance spectrometer (Kea2, Magritek, New Zealand). The microcapillary tubes containing the blood samples are slotted into an impedance matched 1 mm rf-probe tuned at a resonance frequency of 21.57 MHz. The rf-probe is subsequently placed inside a ~0.5 T permanent magnet (Metrolab Instruments, Switzerland) and connected to the bench-top spectrometer for proton ^1^H MRR measurements (Fig. 1(b)). The *R*_2_ relaxation rates are measured using standard Carr-Purcell-Meiboom-Gill (CPMG) pulse sequence with 5000 echoes, 200 μs inter-echo time, transmitter power output of 6.25 mW, and 6 μs 90°-pulse length. A total of 24 scans are acquired for signal averaging and the system is maintained at 26 °C via an external temperature controller. The *R*_2_ measurement of each blood sample is repeated 5–10 times with independent microcapillary tubes.

## Additional Information

**How to cite this article**: Kong, T.F. *et al*. Enhancing malaria diagnosis through microfluidic cell enrichment and magnetic resonance relaxometry detection. *Sci. Rep*. **5**, 11425; doi: 10.1038/srep11425 (2015).

## Figures and Tables

**Table 1:** Properties of healthy and infected red blood cells at three different stages of infection (ring, trophozoite and schizont) in terms of its net volume magnetic susceptibility relative to water^47^, Δχ, density^48^, ρ, and elastic modulus^27^, λ.

	Δχ (10^−6^)	ρ (g cm^−1^)	λ (μN m^−1^)
**Healthy**	−0.18	1.110	8
**Ring**	0.82	1.110	16
**Trophozoite**	0.91	1.106	21.3
**Schizont**	1.80	1.090	53.3

## References

[b1] World Health Organization. Malaria fact sheet. (2014) Available at: http://www.who.int/mediacentre/factsheets/fs094/en/. (Accessed: 2nd December 2014)

[b2] World Health Organization. World malaria report 2013. (2013) Available at: http://www.who.int/malaria/publications/world_malaria_report_2013/en/. (Accessed: 2^nd^ December 2014)

[b3] Sullivan D. J. Hemozoin: a Biocrystal Synthesized during the Degradation of Hemoglobin. Biopolymers Online. 9, 129–137 (2005).

[b4] Center for Disease Control & Prevention. Malaria. (2012) Available at: http://www.cdc.gov/malaria/diagnosis_treatment/treatment.html. (Accessed: 2^nd^ December 2014)

[b5] Tangpukdee N., Duangdee C., Wilairatana P. & Krudsood S. Malaria diagnosis: a brief review. Korean J. Parasitol. 47, 93–102 (2009). 19488414 10.3347/kjp.2009.47.2.93 PMC2688806

[b6] Getahun A., Deribe K. & Deribew A. Determinants of delay in malaria treatment-seeking behaviour for under-five children in south-west ethiopia: a case control study. Malaria J. 9, (2010). 10.1186/1475-2875-9-320 PMC2988828 21070644

[b7] Dondorp A. M. . Artemisinin resistance in plasmodium falciparum malaria. N. Engl. J. Med. 361, 455–467 (2009). 19641202 10.1056/NEJMoa0808859 PMC3495232

[b8] Ashley E. A. . Spread of artemisinin resistance in plasmodium falciparum malaria. N. Engl. J. Med. 371, 411–423 (2014). 25075834 10.1056/NEJMoa1314981 PMC4143591

[b9] Bharti A. R. . Short report: polymerase chain reaction detection of plasmodium vivax and plasmodium falciparum DNA from stored serum samples: implications for retrospective diagnosis of malaria. Am. J. Trop. Med. Hyg. 77, 444–446 (2007). 17827357

[b10] Iqbal J., Sher A., Hira P. R. & Al-Owaish R. Comparison of the optimal test with PCR for diagnosis of malaria in immigrants. J. Clin. Microbiol. 39, 3644–3646 (1999). 10523567 10.1128/jcm.37.11.3644-3646.1999 PMC85714

[b11] Moody A. Rapid diagnostic tests for malaria parasites. Clin. Bio. Rev. 15, 66–78 (2002). 10.1128/CMR.15.1.66-78.2002 PMC118060 11781267

[b12] Kawamoto F. . Sequence variation in the 18s rrna gene, a target for pcr-based malaria diagnosis, in plasmodium ovale from southern vietnam. J. Clin. Microbiol. 34, 2287–2289 (1996). 8862600 10.1128/jcm.34.9.2287-2289.1996 PMC229233

[b13] Morassin B., Fabre R., Berry A. & Magnaval J. One year’s experience with the polymerase chain reaction as a routine method for the diagnosis of imported malaria. Am. J. Trop. Med. Hyg. 66, 503–508 (2002). 12201583 10.4269/ajtmh.2002.66.503

[b14] Lee H., Sun E., Ham D. & Weissleder R. Chip-nmr biosensor for detection and molecular analysis of cells. Nat. Med. 14, 869–874 (2008). 18607350 10.1038/nm.1711 PMC2729055

[b15] Haun J. B. . Micro-nmr for rapid molecular analysis of human tumor samples. Sci. Transl. Med. 3, 71ra16 (2011). 10.1126/scitranslmed.3002048 PMC3086073 21346169

[b16] Issadore D. . Miniature magnetic resonance system for point-of-care diagnostics. Lab Chip 11, 2282–2287 (2011). 21547317 10.1039/c1lc20177h PMC3115660

[b17] Peng W. K., Chen L. & Han J. Development of miniaturized, portable magnetic resonance relaxometry system for point-of-care medical diagnosis. Rev. Sci. Instrum. 83, 095115 (2012). 23020427 10.1063/1.4754296

[b18] Massin C. . High-q factor rf planar microcoils for micro-scale nmr spectroscopy. Sens. Actuators A Phys. 97-98, 280–288 (2002).

[b19] Hsieh C. Y., Yeh Y. T. & Fan L. S. Multilayer high-aspect-ratio rf coil for nmr applications. Microsyst. Technol. 17, 1311–1317 (2011).

[b20] Peng W. K. . Micro magnetic resonance relaxometry for label-free, rapid malaria diagnosis. Nat. Med. 20, 1069–1073 (2014). 25173428 10.1038/nm.3622

[b21] Gascoyne P. . Microsample preparation by dielectrophoresis: isolation of malaria. Lab Chip 2, 70–75 (2002). 15100837 10.1039/b110990c PMC2726252

[b22] Shafiee H., Caldwell J. L., Sano M. B. & Davalos R. V. Contactless dielectrophoresis: a new technique for cell manipulation. Biomed. Microdevices 11, 997–1006 (2009). 19415498 10.1007/s10544-009-9317-5

[b23] Ribaut C. . Concentration and purification by magnetic separation of the erythrocytic stages of all human plasmodium species. Malaria J. 7, (2008). 10.1186/1475-2875-7-45 PMC2292734 18321384

[b24] Zimmerman P. A., Thomson J., Fujioka H., Collins W. & M. Z. Diagnosis of malaria by magnetic deposition microscopy. Am. J. Trop. Med. Hyg. 74, 568–572 (2006). 16606985 PMC3728894

[b25] Hou H. W. . Deformability based cell margination–a simple microfluidic design for malaria-infected erythrocyte separation. Lab Chip 10, 2605–2613 (2010). 20689864 10.1039/c003873c

[b26] Cheng R., Lai Y. G. & Chandran K. B. Three-dimensional fluid-structure interaction simulation of bileaflet mechanical heart valve flow dynamics. Ann. Biomed. Eng. 32, 1471–1483 (2004). 15636108 10.1114/b:abme.0000049032.51742.10 PMC1404505

[b27] Suresh S. . Connections between single-cell biomechanics and human disease states: gastrointestinal cancer and malaria. Acta Biomater. 1, 15–30 (2005). 16701777 10.1016/j.actbio.2004.09.001

[b28] Pierigè F., Serafini S., Rossi L. & Magnani M. Cell-based drug delivery. Adv. Drug Deliv. Rev. 60, 286–295 (2008). 17997501 10.1016/j.addr.2007.08.029

[b29] Silva D. C. N. . Optical tweezers as a new biomedical tool to measure zeta potential of stored red blood cells. PLoS ONE 7, e31778 (2012). 22363729 10.1371/journal.pone.0031778 PMC3283675

[b30] Gottlieb Y. . Physiologically aged red blood cells undergo erythrophagocytosis *in vivo* but not *in vitro*. Haematologica 97, 994–1002 (2012). 22331264 10.3324/haematol.2011.057620 PMC3396668

[b31] Mendanha S. A., Anjos J. L. V., Silva A. H. M., & Alonso A. Electron paramagnetic resonance study of lipid and protein membrane components of erythrocytes oxidized with hydrogen peroxide. Braz. J. Med. Biol. Res. 45, 473–481 (2012). 22473321 10.1590/S0100-879X2012007500050 PMC3854297

[b32] Rettig M. P. . Evaluation of biochemical changes during *in vivo* erythrocyte senescence in the dog. Blood 93, 376–384 (1999). 9864184

[b33] Umbreit J. Methemoglobin–it’s not just blue: a concise review. Am. J. Hematol. 82, 134–144 (2007). 16986127 10.1002/ajh.20738

[b34] Kanias T. & Acker J. P. Biopreservation of red blood cells–the struggle with hemoglobin oxidation. FEBS J. 277, 343–356 (2010). 19968714 10.1111/j.1742-4658.2009.07472.x

[b35] Low P. S., Waugh S. M., Zinke K. & Drenckhahn D. The role of hemoglobin denaturation and band 3 clustering in red blood cell aging. Science 227, 531–533 (1985). 2578228 10.1126/science.2578228

[b36] Li X. & Zhou Y. Microfluidic Devices for Biomedical Applications [303–306] (Woodhead Publishing, Cambridge, 2013).

[b37] Shevkoplyas S. S., Yoshida T., Munn L. L. & Bitensky M. W. Biomimetic autoseparation of leukocytes from whole blood in a microfluidic device. Anal. Chem. 77, 933–937 (2005). 15679363 10.1021/ac049037i PMC3022340

[b38] Kumar A. & Graham M. D. Margination and segregation in confined flows of blood and other multicomponent suspensions. Soft Matter 8, 10536–10548 (2012). 10.1103/PhysRevLett.109.108102 23005332

[b39] Goldsmith H. L., Cokelet G. R. & Gaehtgens P. Robin fahraeus: evolution of his concepts in cardiovascular physiology. Am. J. Physiol. 257, H1005–H1015 (1989). 2675631 10.1152/ajpheart.1989.257.3.H1005

[b40] Zhang J., Li W., Li M., Alici G. & Nguyen N.-T. Particle inertial focusing and its mechanism in a serpentine microchannel. Microfluid. Nanofluid. 17, 305–316 (2014).

[b41] Zhao H., Shaqfeh E. S. G. & Narsimhan V. Shear-induced particle migration and margination in a cellular suspension. Phys. Fluids 24, 011902 (2012).

[b42] Zhang J., Yan S., Li W., Alici G. & Nguyen N.-T. High throughput extraction of plasma using a secondary flow-aided inertial microfluidic device. RSC Adv. 4, 33149–33159 (2014).

[b43] Eaton P., Zuzarte-Luis V., Mota M. M., Santos N. C. & Prudłncio M. Infection by plasmodium changes shape and stiffness of hepatic cells. Nanomed. Nanotechnol. 8, 17–19 (2012). 10.1016/j.nano.2011.10.004 22033078

[b44] Chen Y. . Rare cell isolation and analysis in microfluidics. Lab Chip 14, 626–645 (2014). 24406985 10.1039/c3lc90136j PMC3991782

[b45] Glenister F. K., Coppel R. L., Cowman A. F., Mohandas N. & Cooke B. M. Contribution of parasite proteins to altered mechanical properties of malaria-infected red blood cells. Blood 99, 1060–1063 (2002). 11807013 10.1182/blood.v99.3.1060

[b46] Moll K., Ljungström I., Perlmann H., Scherf A. & Wahlgren M. Methods in Malaria Research (MR4/ATCC, Virginia, 2013).

[b47] Moore L. R. . Hemoglobin degradation in malaria-infected erythrocytes determined from live cell magnetophoresis. FASEB J. 20, 747–749 (2006). 16461330 10.1096/fj.05-5122fje PMC3728832

[b48] Saul A., Myler P., Elliott T. & Kidson C. Purification of mature schizonts of plasmodium falciparum on colloidal silica gradients. Bull. World Health Organ. 60, 755–759 (1982). 6758971 PMC2536039

